# Brief and Indirect Exposure to Natural Environment Restores the Directed Attention for the Task

**DOI:** 10.3389/fpsyg.2021.619347

**Published:** 2021-07-07

**Authors:** Tsukasa Kimura, Tatsuya Yamada, Yohko Hirokawa, Kazumitsu Shinohara

**Affiliations:** ^1^The Institute of Scientific and Industrial Research, Osaka University, Ibaraki, Japan; ^2^School of Human Sciences, Osaka University, Suita, Japan; ^3^Graduate School of Human Sciences, Osaka University, Suita, Japan

**Keywords:** natural environment, restorative environment, attention restoration, directed attention, SCL

## Abstract

The mental fatigue elicited by working and studying consumed mental resources, thereby eliciting a declined performance and an increased mental stress. The long-term continuous work and study, which is typical for modern workers and students, can increase mental fatigue and health risks. Previous studies reported that the natural environment (i.e., forest and waterside) has a restorative of mental resources (i.e., attention) and reducing stress. However, it is difficult for urban workers and students to take sufficient breaks in real natural environment. We conducted an experiment to examine whether brief and indirect exposure to the natural environment elicits a restorative of attention and reducing stress. Twenty-five undergraduate and graduate students from the university of modern city participated in the experiment. The experiment involved measuring the changes in the task performance of the participants (i.e., sustained attention to response task) and the subjective mental workload (i.e., arousal, valence, and NASA-TLX), while the attention restoration was indexed from physiological response (i.e., skin conductance level, SCL) over time. The participants had two types of resting periods in the middle of the task, i.e., by looking at a blank display (simple break) or by watching a nature video having scenes of, e.g., a forest, small waterfall, and rustling leaves (nature break). The results indicate that the natural environment indirectly depicted through the nature videos does not affect the task performance and the subjective mental workload but decreases the SCL. The results of the physiological response suggest that having rest periods depicting the natural environment, even if indirectly and briefly, can restore the directed attention (i.e., mental resources) for the task. This experiment revealed a useful method of resting for urban workers and students to restore their attention to a task.

## Introduction

There are many stress factors in our environment. For example, the mental fatigue elicited by working and studying consumed mental resources, thereby eliciting a declined performance and an increased mental stress (e.g., Kaplan and Kaplan, 1989; Staats et al., 2003). Long-term continuous work and study, which is typical for modern workers and student, can increase mental fatigue and health risks (e.g., Selye, 1956), and therefore, it is necessary to reduce these negative effects. Efficient stress-reducing techniques have been used for attracting attention in recent years.

Previous studies reported that the natural environment has a restoration for attention and reducing stress (for a review, see Berto, 2014). This effect is explained with two representative frameworks, i.e., Attention Restoration Theory (ART; Kaplan and Kaplan, 1989; Kaplan, 1995; Kaplan and Berman, 2010) and Stress Recovery Theory (SRT; Ulrich, 1983; Ulrich et al., 1991). ART focuses on directed attention when we concentrate on an object or event. When we concentrate on performing a task for a long time, directed attention, which is a limited cognitive resource, deteriorates over time. This deterioration of attention is closely related to decreased task performance (e.g., Fortenbaugh et al., 2017). To maintain a certain degree of task performance, it is necessary to effectively restore directed attention. In ART, it is thought that the natural environment separates the attention from the task and stops the consumption of attention directed to tasks, thereby restoring the attentional resource (Kaplan and Kaplan, 1989; Kaplan, 1995; Kaplan and Berman, 2010). SRT focuses on the relationship between natural environment and reduction of mental stress. In SRT, it is thought that the natural environment is related to food, water, and housing. The humans evolved over a long period in natural environment and the factors in environment functioned as favorable ones for human survival during evolution. Therefore, the natural environment is thought to provide a reducing response for physiological stress (Ulrich, 1983; Ulrich et al., 1991). However, it is unclear what internal process is activated when coming into contact with the natural environment to recover cognitive resources and promote relaxation (e.g., Berto, 2014; Joye and Dewitte, 2018).

To examine these natural environment effects, the measures of stress, mental workload, and directed attention for the task are needed. The main methods of measuring stress and mental workload are subjective ratings and physiological responses. For example, NASA Task Load Index (NASA-TLX) is the most commonly used subjective rating for measuring stress and mental workload (Hart and Staveland, 1988). It is also used to examine the relationship between attention resources and mental workload (e.g., Temple et al., 2000; Bunce and Sisa, 2002). The change of skin resistance caused by sweating is often used to examine the attention and mental workload. Sweating is facilitated via sympathetic activity; it is possible to measure various psychological activities using the skin conductance level (SCL) calculated from sweating. SCL is the inverse of skin resistance, i.e., it reflects sympathetic activity (e.g., Dawson et al., 2007; Boucsein et al., 2012), attention for the task, and mental workload caused by consumption of attention (e.g., Frith and Allen, 1983; Foy and Chapman, 2018). Moreover, it is also known that SCL decreases in response to the restoration of attention by the natural environment (e.g., Hedblom et al., 2019). As a method for the measurement of directed attention for the task, sustained attention to response task (SART) is often used. The SART involves presenting various digits on a display, and participants are instructed to respond with key press as quickly and accurately as possible to the digits with each digit exception of the digit “3,” which required response inhibition. The SART performance decreases over time, and it is thought that this result reflects decreasing the attention of the participants for the task (Robertson et al., 1997). Moreover, SART is often used to examine whether the natural environment restores attention (e.g., Berto, 2005; Lee et al., 2015; Pasanen et al., 2018).

Previous studies reported that experiencing the natural environment is useful to restore attentional resource and reduce stress (for a review, see Berto, 2014); however, it is difficult for urban workers and students to experience the real natural environment in their everyday life. Previous studies have also reported that images and videos of the natural environment (i.e., virtual natural environment) have a restorative effect (e.g., Berto, 2005; Berto et al., 2010; Emfield and Neider, 2014). Also this virtual natural environment decreased SCL (e.g., Hedblom et al., 2019). Therefore, it is possible that natural environment can have a restorative effect, even if it is depicted virtually and indirectly. Recent studies reported that even brief exposure to the natural environment has such an effect (e.g., Lee et al., 2015; Bourrier et al., 2018). Taken together, it is possible that this effect can also be elicited by indirect and brief exposure. This effect is beneficial for urban workers and students.

To test this hypothesis, we conducted an experiment to measure the changes in the task performance of the participants, subjective mental workload, and physiological response over time as indices of the restorative effect and manipulated how the participants took breaks during the experiment. The participants took two breaks during the experiment. In each break, participants experienced either the simple break or the nature break. During the simple break, participants looked at a blank display. During the nature break, participants watched a nature video including scenes of, e.g., a forest, small waterfall, and rustling leaves. All participants experienced both breaks. The SART was used to measure the directed attention for the task before and after the break. We predicted that the performance of this task after taking nature breaks would be higher than after taking simple breaks because the natural environment facilitates the restoration of directed attention. The questionnaire regarding arousal and valence (Russell, 1980), and the Japanese version of NASA-TLX (Haga and Mizukami, 1996) were used as indices of subjective mental workload. Kimura et al. (2020) found that a sustained task can increase stress and mental workload even if work efficiency is maintained. We predicted that mental workload after taking nature breaks would decrease compared with after taking simple breaks, assuming the natural environment has a stress-reducing effect. In addition, SCL during the experiment is used as the index of sympathetic activity. Typically, the SCL increases during a task compared with a rest session and then decreases after the task ends. In this context, it is thought that sympathetic activity increases during the task period due to the attention on the task. After the task, this attention is restored (e.g., Dawson et al., 2007; Boucsein et al., 2012). Therefore, we predicted that the SCL during the SART session would be higher than during a rest session before the task and the simple and natural breaks taken in the middle of the task. We also predicted that the SCL during nature breaks would be lower than during simple breaks, assuming that the natural environment restores the attention from the task.

## Materials and Methods

### Participants

Thirty undergraduate and graduate students (15 females and 15 males; 20–29 years of age) from the University of modern city participated in the experiment. All participants were right-handed according to their self-report and had normal or corrected-to-normal vision. This experiment was approved by the Behavioral Research Ethics Committee of the Osaka University School of Human Sciences. Written informed consent was obtained from all participants, and their rights as experimental subjects were protected. The data from five participants had to be excluded (due to recording problems with the behavioral data, subjective rating data, and physiological data, and noise with SCL value over 20 μS), resulting in the data collected from 25 participants (13 females and 12 males; 20–29 years of age). We conducted a power analysis using the R and the pwr package (Champely et al., 2018). In this sample size, the effect size for the two-way interaction between two conditions and three sessions on ΔSCL was *f* = 0.26 at significance level of α = 0.05 and power of 1 – β = 0.80, which was a medium effect (Cohen, 1992).

### Stimulus and Equipment

#### Sustained Attention to Response Task

Digits were presented randomly from “1” to “9.” The visual angle of one digit was 3.82 by 2.86 from an observing distance of 60 cm. All digits were presented centrally in white against a black background.

#### Subjective Rating

Arousal and valence (Russell, 1980) were measured on a 9-point scale (from 1 to 9). A low score means low arousal and unpleasantness. Moreover, five items from the Japanese version of NASA-TLX (Hart and Staveland, 1988; Haga and Mizukami, 1996) were measured on a 10-point scale (from 0 to 9) and consisted of mental demand, performance, effort, frustration, and overall mental workload. A low score means low mental demand, effort, frustration, and overall mental workload. A low score only in performance means high performance, because it is an inverted scale.

#### Nature Videos

Two nature videos (Stimulus ID: https://www.landskip.jp/movies/759; https://www.landskip.jp/movies/895), provided by LandSkip Inc. (https://www.landskip.jp/), were used as natural stimuli. These videos were selected based on the previous results indicating the restorative factor of environment, e.g., forest and waterfront (Laumann et al., 2001). Both videos were recorded in 4K resolution and included scenes of a forest, autumn leaves, small waterfall, river, and sounds of flowing water and rustling leaves.

#### Equipment

The presentation of stimuli, scales, and instructions was controlled with MATLAB R2010b (MathWorks Inc, U.S.A.) and Psychtoolbox (Kleiner et al., 2007) installed on a desktop computer (STYLE-R027-i7-HN, iiyama). A 21.5-inch LCD monitor (XU2290HS-B2, iiyama) was placed on the desk to present these stimuli, scales, and instructions. The nature videos were presented using a 65-inch LED monitor (JN-V6500UHDR, JAPANNEXT; resolution: 3840 by 2160) placed 170 cm behind the participant.

#### Recording of SCL

The SCL data were recorded using an electrodermal activity (EDA) amplifier MaP1720CA and EDA unit APU030 (Nihonsanteku) using disposable electrodes (Mets Inc.). These electrodes were placed on the left index and middle fingers. These data were recorded using InputMonitor software (Nihonsanteku), and the sampling rate was 500 Hz and the bandpass filter was 0–15 Hz.

#### Procedure

**Figure 1:**
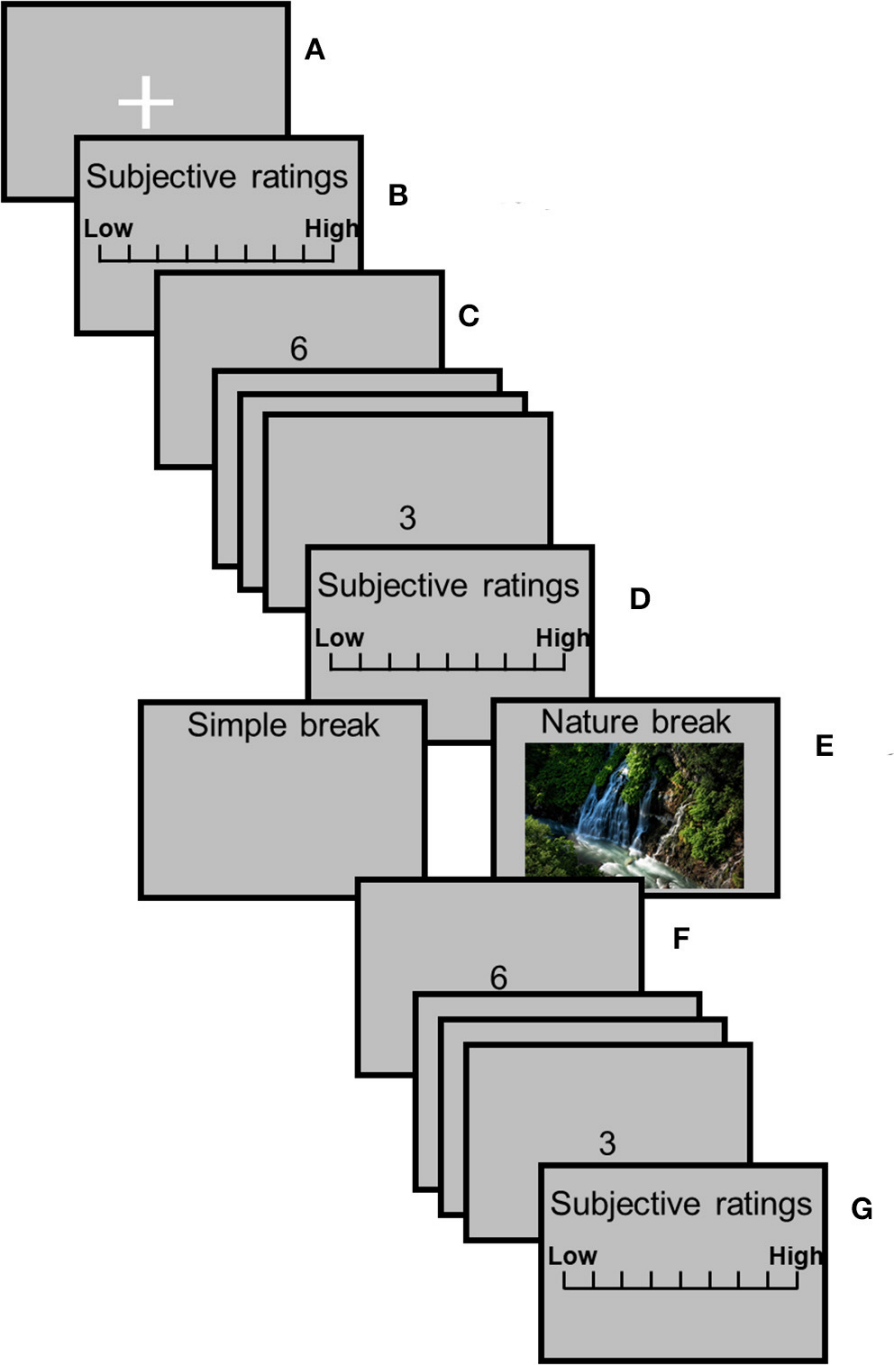
Experimental procedure. **(A)** Rest session; **(B,D,G)** subjective rating sessions; **(C,F)** pre-recovery SART and post-recovery SART sessions; and **(E)** recovery session.

Figure 1 illustrates the experimental procedure. This experiment involved one rest session [i.e., (a)], three subjective rating sessions [i.e., (b), (d), and (g)], two SART sessions [i.e., (c) pre-recovery and (f) post-recovery], and one recovery session [i.e., (e) one of the two breaks]. In the experimental room, participants sat in a chair with the electrodes placed on their fingers to record the SCL. They were asked not to move more than necessary to avoid artifacts in the physiological data. During the rest session, participants were asked to gaze at a white fixation cross (visual angle of 2.86 by 2.86 from an observing distance of 60 cm) in the center of a gray background for 120 s. After the rest session, participants responded to a subjective questionnaire using a keyboard during the subjective rating session. After this session, the pre-recovery SART session began. During this session, each digit was presented for 200 ms, and the stimulus onset asynchrony (SOA) was 1,200 ms. Participants were instructed to respond as quickly and accurately as possible to the digits by pressing the space key on presentation of each digit (go-trials) with their right index finger with the exception of digit “3,” which required response inhibition. Stimuli were presented 500 times in one session (“3” was presented 50 times, i.e., 10%). One session took ~10 min. Participants then responded to the subjective questionnaire again and had a recovery session involving a 5 min simple or nature break. During the simple break, participants turned their chairs around and were instructed to look at the blank display. During the nature break, participants were instructed to turn their chairs around and watch a nature video. The participants watched one of the two nature videos. Which of the two nature videos to present were counterbalanced among the participants. After the recovery session, participants performed the post-recovery SART session, and they responded to the subjective questionnaire again. After all the sessions, participants took a 3-h break and performed all the sessions again under another recovery condition, i.e., all participants experienced both conditions. The order of conditions was randomized between participants.

#### Data Analysis

To analyze the behavioral data from the SART sessions, RTs for correct answer and hit rate were calculated for each session. A “hit” in the SART means that a participant correctly responded to the appearance of a digit, except “3.” Subjective rating data were analyzed using questionnaire scores. The SCL data were averaged for each session. We also subtracted the average SCL of the rest session from that of each session, i.e., we calculated the ΔSCL of pre-recovery SART, recovery, and post-recovery SART sessions.

Two-way repeated measures ANOVA on RTs and hit rate were conducted under two conditions (simple and nature break conditions) and two SART sessions (pre-recovery and post-recovery). Moreover, a two-way ANOVA on the result of subjective rating was conducted under two conditions and three sessions (rest, pre-recovery, and post-recovery SART sessions). Furthermore, a two-way ANOVA on ΔSCL was conducted under two conditions and three sessions (pre-recovery SART session, recovery session, and post-recovery SART session). These ANOVAs were conducted by applying Greenhouse–Geisser corrections to the degrees of freedom when appropriate (Greenhouse and Geisser, 1959). The effect sizes were indicated in terms of partial eta squared (ηp^2^). *Post hoc* comparisons were made using Shaffer's modified sequentially rejective multiple test procedure, which extends the Bonferroni *t*-test in a stepwise fashion (Shaffer, 1986). The significance level was set at *p* < 0.05 for all statistical analyses.

## Results

### Behavioral Data

**Figure 2:**
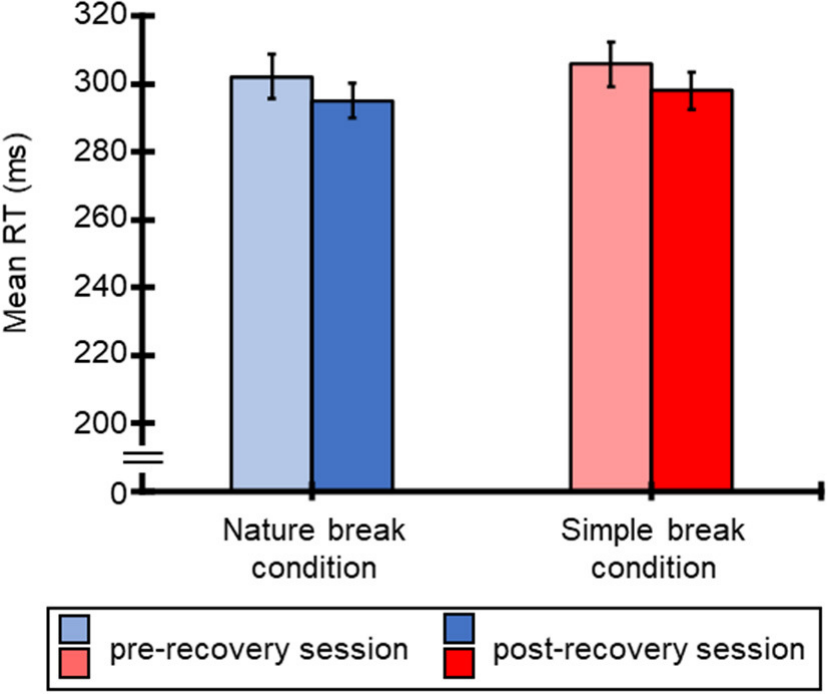
Mean RTs (ms) for SART and standard errors of RTs, regarding pre-recovery and post-recovery sessions in both conditions.

Figure 2 shows the mean RTs for the SART for all participants for each break condition. Averaged RTs of all participants were 302 ms (SE = 0.007), 295 ms (SE = 0.005), 306 ms (SE = 0.007), and 298 ms (SE = 0.006) for the nature break-pre-recovery, nature break-post-recovery, simple break-pre-recovery, and simple break-post-recovery conditions. Table 1 shows the results of ANOVA for these RTs. The ANOVA for the mean RTs revealed the main effect of session, and this result means that the RTs for the post-recovery SART session was shorter than those for the pre-recovery SART session. However, the main effect of condition and interaction were not significant.

**Table 1:** Results of ANOVA for RTs.

**Interaction and main effect**	***F***	***df***	***p***	**$\eta_{\text{p}}^{2}$**
Conditions × sessions	0.02	1, 24	0.90	0.01
Conditions	0.66	1, 24	0.42	0.03
Sessions	9.66	1, 24	0.005^**^	0.29

^*^p < 0.05,

** *p < 0.01, and*

^***^p < 0.001.

**Figure 3:**
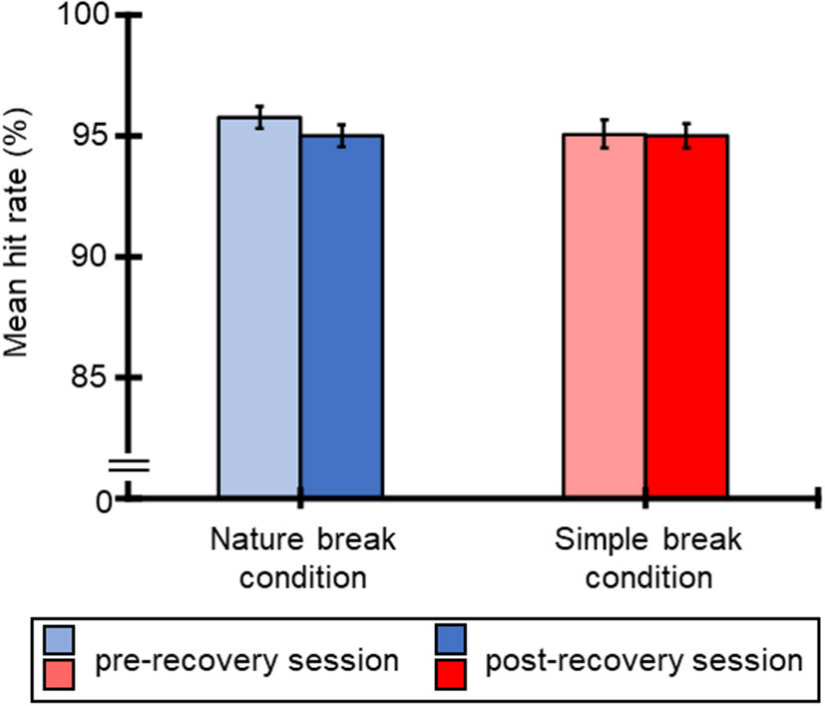
Mean hit rate (%) for SART and standard errors of RTs, regarding pre-recovery and post-recovery sessions in both conditions.

Figure 3 illustrates the mean hit rate for the SART for all participants for each break condition. Averaged RTs of all participants were 95.8% (SE = 0.45), 95.0% (SE = 0.45), 95.1% (SE = 0.56), and 95.0% (SE = 0.51) for the nature break-pre-recovery, nature break-post-recovery, simple break-pre-recovery, and simple break-post-recovery conditions. Table 2 shows the results of ANOVA for these hit rates. The ANOVA for the mean hit rate indicated that all main effects and interaction were not significant.

**Table 2:** Results of ANOVA for hit rates.

**Interaction and main effect**	***F***	***df***	***p***	**$\eta_{\text{p}}^{2}$**
Conditions × sessions	1.19	1, 24	0.29	0.05
Conditions	0.65	1, 24	0.43	0.03
Sessions	1.92	1, 24	0.18	0.07

^*^p < 0.05, ^**^p < 0.01, and ^***^p < 0.001.

### Subjective Rating

**Figure 4:**
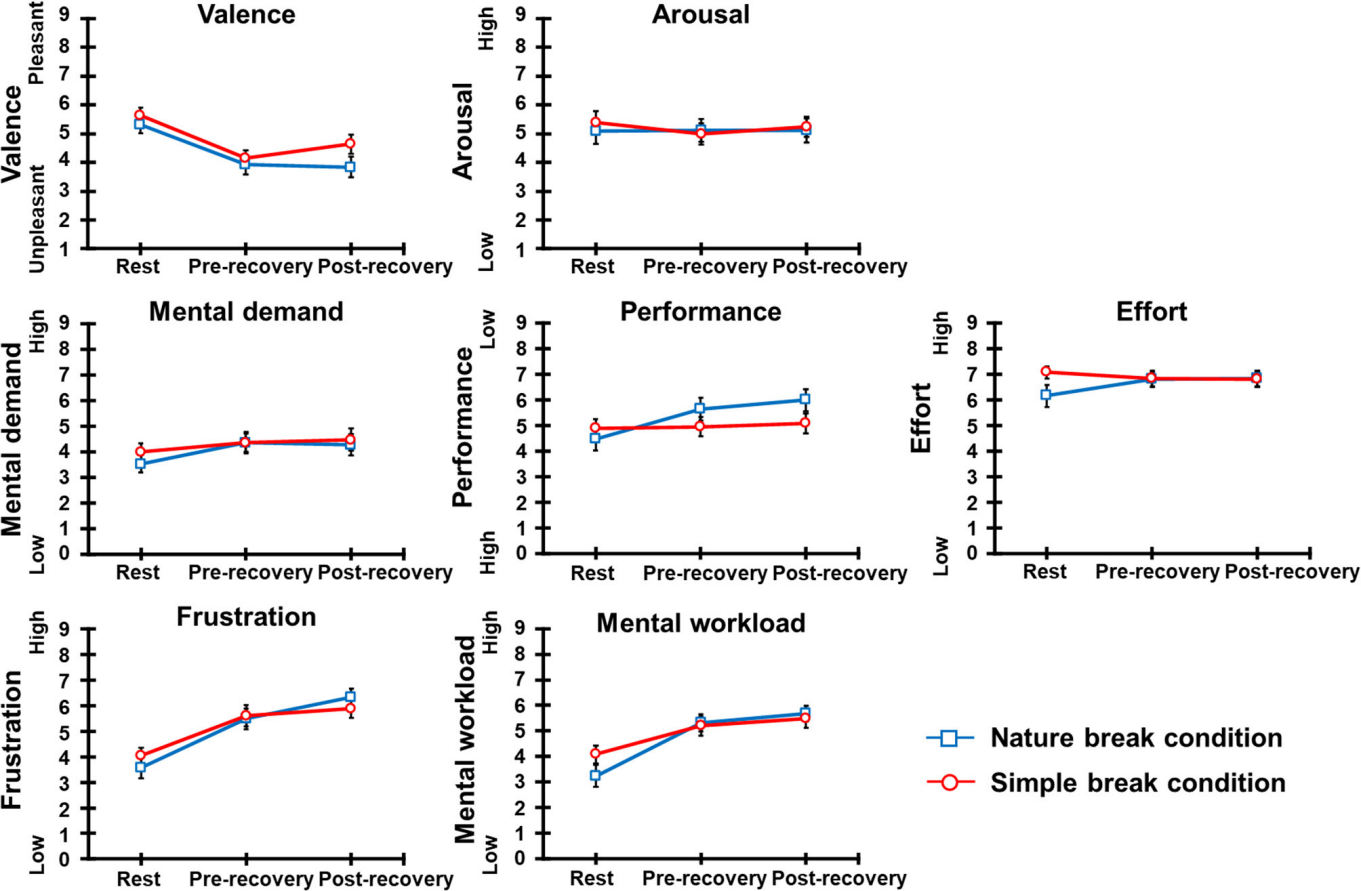
Subjective ratings and standard errors for each simple and nature break conditions.

Figure 4 illustrates the mean subjective ratings for all participants for each break condition, and Table 3 shows the results of ANOVA for these subjective ratings.

**Table 3:** Results of ANOVA for subjective ratings.

**Interaction and main effect for each item**	***F***	***df***	***p***	**$\eta_{\text{p}}^{2}$**
**Valence**				
Conditions × sessions	0.88	2, 48	0.40	0.04
Conditions	3.51	1, 24	0.07	0.13
Sessions	17.29	2, 48	<0.001^***^	0.42
**Arousal**				
Conditions × sessions	0.45	2, 48	0.64	0.02
Conditions	0.14	1, 24	0.71	0.01
Sessions	0.16	2, 48	0.82	0.01
**Mental demand**				
Conditions × sessions	0.78	2, 48	0.45	0.03
Conditions	1.28	1, 24	0.27	0.05
Sessions	5.20	2, 48	0.02^*^	0.18
**Performance**				
Conditions × sessions	2.32	2, 48	0.11	0.09
Conditions	1.64	1, 24	0.21	0.06
Sessions	3.40	2, 48	0.06	0.12
**Effort**				
Conditions × sessions	3.18	2, 48	0.07	0.12
Conditions	3.12	1, 24	0.09	0.12
Sessions	0.51	2, 48	0.53	0.02
**Frustration**				
Conditions × sessions	2.96	2, 48	0.07	0.11
Conditions	0.08	1, 24	0.78	0.01
Sessions	31.63	2, 48	<0.001^***^	0.57
**Overall mental workload**				
Conditions × sessions	3.84	2, 48	0.04^*^	0.14
Conditions	0.70	1, 24	0.41	0.03
Sessions	27.35	2, 48	<0.001^***^	0.53

* *p < 0.05*,

^**^p < 0.01, and

*** *p < 0.001*.

In the valence, the main effect of session was significant, and *post hoc* comparisons indicated that valence after the pre-recovery and post-recovery SART sessions was lower (i.e., unpleasant) than after the rest session. The main effect of condition and interaction were not significant. In the arousal, all main effects and interaction were not significant.

In the mental demand, the main effect of session was significant, and *post hoc* comparisons indicated that mental demand after the pre-recovery and post-recovery SART sessions was higher (i.e., high load) than after the rest session. The main effect of condition and interaction were not significant. In the performance and effort, all main effects and interaction were not significant. In the frustration, the main effect of session was significant, and *post hoc* comparisons indicated that frustration after the pre-recovery and post-recovery SART sessions were higher (i.e., high frustration) than after the rest session. The main effect of condition and interaction were not significant. In the overall mental workload, the interaction was significant, and *post hoc* comparisons indicated that the overall mental workload after the pre-recovery and post-recovery SART sessions were higher (i.e., high load) than after the rest session for each break condition. Moreover, the main effect of session was significant, and *post hoc* comparisons indicated that the overall mental workload was higher after the pre-recovery SART session than after the rest session and higher after the post-recovery SART session than after the pre-recovery SART session. The main effect of condition was not significant.

### Skin Conductance Level

**Figure 5:**
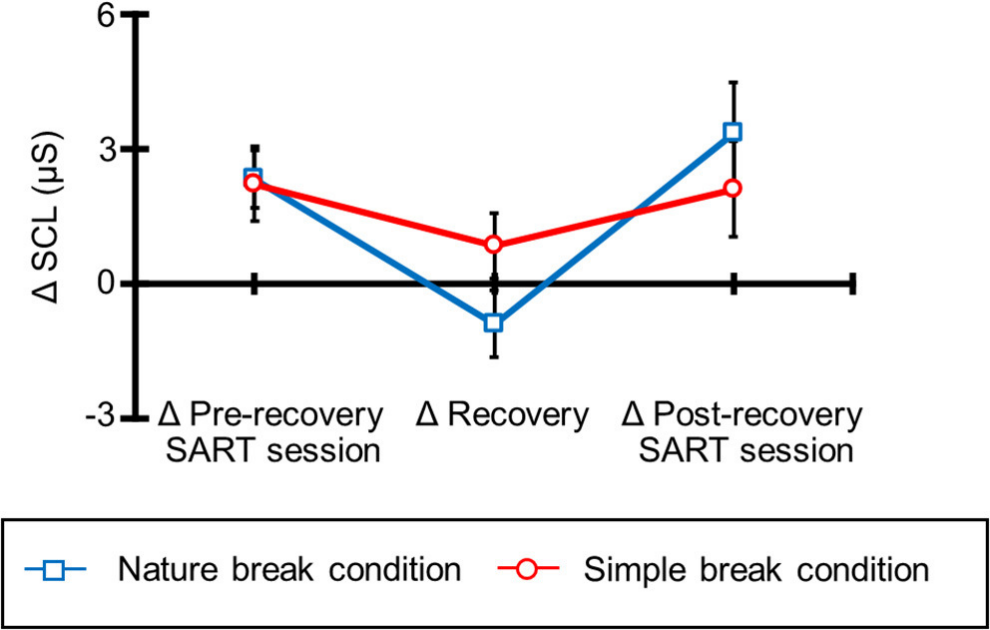
ΔSCL and standard error of ΔSCL for each simple and nature break conditions.

Figure 5 shows the mean ΔSCL for all participants under each break condition. The average ΔSCL values of all participants were 2.32 (SE = 0.65), −8.99 (SE = 0.74), 3.36 (SE = 1.13), 2.22 (SE = 0.83), 0.83 (SE = 0.73), and 2.10 (SE = 1.07) for the nature break-pre-recovery, nature break-recovery, nature break-post-recovery, simple break-pre-recovery, simple break-recovery, and simple break-post-recovery conditions. Table 4 shows the results of ANOVA for these ΔSCL. The ANOVA revealed the interaction, and *post hoc* comparisons indicated that the ΔSCL was lower during the recovery session than during the pre-recovery SART and post-recovery SART sessions in the nature break condition. Moreover, the main effect of session was significant, and *post hoc* comparisons indicated that the ΔSCL was lower during the recovery session than during the pre-recovery SART and post-recovery SART sessions. The main effect of condition was not significant.

**Table 4:** Results of ANOVA for SCL.

**Interaction and main effect**	***F***	***df***	***p***	**$\eta_{\text{p}}^{2}$**
Conditions × sessions	3.31	2, 48	0.04^*^	0.12
Conditions	0.02	1, 24	0.88	0.01
Sessions	5.40	2, 48	0.01^**^	0.18

* *p < 0.05*,

** *p < 0.01, and*

^***^p < 0.001.

## Discussion

We investigated whether brief and indirect exposure to the natural environment elicits a restorative effect and reducing stress. To test this hypothesis, we conducted an experiment to measure the task performance, subjective mental workload, and physiological response over time when performing the task included simple or nature breaks.

As indicated in Figure 2, the mean RTs for the SART of second block (i.e., post-recovery SART session) were shorter than the first block (i.e., pre-recovery SART session), and the hit rate was kept over 90% throughout the task, suggesting that task performance was maintained during the task for both break conditions. However, the subjective unpleasantness (i.e., valence), mental demand, frustration, and overall mental workload increased after the SART for both break conditions. This corresponds to a previous study in which the mental workload was increased by repeating the task, even when the task performance was maintained (Kimura et al., 2020). In summary, the recovery session for both break conditions helped to maintain task performance but did not reduce the mental workload. Although previous studies reported that the directed attention measured during the SART was restored due to the natural environment (e.g., Berto, 2005; Lee et al., 2015; Pasanen et al., 2018), our results indicate that the restorative effect of the natural environment is small for the SART. Considering that this effect is small for certain attention tasks (Ohly et al., 2016), more extensive exposure to the natural environment may be required to restore directed attention consumed by performing the SART. For future research, the relationship among the restorative effect of the natural environment, type of attention manipulated by a task, task time, and task performance should be examined.

The SCL, however, was lowest during the recovery session in a nature break condition. The SCL reflects sympathetic activity and increases during the task period due to the attention on the task (e.g., Dawson et al., 2007; Boucsein et al., 2012). The directed attention during a task should be maintained, and after the task we need to restore this attention by taking breaks to prepare for the next task. Previous studies reported that the natural environment restores the directed attention to objects and events, resulting in the recovery of cognitive resources (Kaplan and Kaplan, 1989; Kaplan, 1995; Kaplan and Berman, 2010). Therefore, our results showed that taking breaks that involve the natural environment (i.e., nature break condition) restore the attention directed at a task and decreases the SCL, like in previous studies. Moreover, this effect also occurred with brief (i.e., 5 min) and indirect (i.e., videos) exposure, unlike in previous studies. This finding will be beneficial for urban workers and students. Our results propose a useful method of resting for urban workers and students to recover the attention.

Finally, several points about these results need to be considered. First, it is necessary to consider the difference in work time between an artificial laboratory setting and real-world settings including working and studying. In previous studies examining the relationship between the natural environment and sustained attention measured by performing SART, the task time was usually about 5–10 min per session (e.g., Berto, 2005; Lee et al., 2015; Pasanen et al., 2018). This is an excellent experimental setting to examine the changes in directed attention over a short period and the recovery effect due to the natural environment, whereas real-world working and studying times are longer than these experimental times, and the time until taking a break is longer. Therefore, it is necessary to examine whether brief and indirect exposure to the natural environment also restores directed attention in more realistic work and study situations. In addition, it is necessary to add a no-break condition in these situations. By adding this condition, we can examine not only the simple effect of repeating the work but also the effects of a break and the type of break adding. It is also unclear about the relationship that exists between the restoration of physiological attention and the subsequent task performance. In our study, the natural environment restored the physiological attention but did not affect the subsequent task performance and subjective mental workload. In the future studies, the causal relationship among restored physiological attention, task performance, and subjective mental workload should be examined to understand the cognitive processes involved in the restorative effect of the natural environment.

## Conclusions

The results of physiological response revealed that breaks involving the natural environment restored the directed attention for the task, even in brief and indirect exposure. This study expanded our understanding of the effect of attention restoration and reducing stress by the natural environment and revealed a useful method of resting for urban workers and students to recover their directed attention.

## Data Availability Statement

The datasets presented in this article are not readily available because these data contains personal information. Requests to access the datasets should be directed to corresponding author. Requests to access the datasets should be directed to Tsukasa Kimura, kimura@ai.sanken.osaka-u.ac.jp.

## Ethics Statement

The studies involving human participants were reviewed and approved by Behavioral Research Ethics Committee of the Osaka University School of Human Sciences. The patients/participants provided their written informed consent to participate in this study.

## Author Contributions

TK, TY, and KS contributed to the conception and design of the study. TK, TY, and YH contributed to the data acquisition. TK wrote the first draft of the manuscript. All authors contributed to the statistical analysis and discussing, revising, and reading the manuscript and finally approved the submitted version.

## Conflict of Interest

The authors declare that the research was conducted in the absence of any commercial or financial relationships that could be construed as a potential conflict of interest.
